# Scalable production of recombinant three-finger proteins: from inclusion bodies to high quality molecular probes

**DOI:** 10.1186/s12934-024-02316-1

**Published:** 2024-02-12

**Authors:** Jiang Xu, Xiao Lei, Ao Li, Jun Li, Shuxing Li, Lin Chen

**Affiliations:** 1 Molecular and Computational Biology, Department of Biological Sciences, University of Southern California, Los Angeles, CA 90089 USA; 2 Institute of Clinical Medicine, The First Affiliated Hospital of University of South China, Hengyang, Hunan 421001 People’s Republic of China

**Keywords:** Three finger protein, *E. coli* recombinant expression, Inclusion body refolding, Disulfide bond formation, Oxidation refolding

## Abstract

**Background:**

The three-finger proteins are a collection of disulfide bond rich proteins of great biomedical interests. Scalable recombinant expression and purification of bioactive three-finger proteins is quite difficult.

**Results:**

We introduce a working pipeline for expression, purification and validation of disulfide-bond rich three-finger proteins using *E. coli* as the expression host. With this pipeline, we have successfully obtained highly purified and bioactive recombinant α-Βungarotoxin, k-Bungarotoxin, Hannalgesin, Mambalgin-1, α-Cobratoxin, MTα, Slurp1, Pate B etc. Milligrams to hundreds of milligrams of recombinant three finger proteins were obtained within weeks in the lab. The recombinant proteins showed specificity in binding assay and six of them were crystallized and structurally validated using X-ray diffraction protein crystallography.

**Conclusions:**

Our pipeline allows refolding and purifying recombinant three finger proteins under optimized conditions and can be scaled up for massive production of three finger proteins. As many three finger proteins have attractive therapeutic or research interests and due to the extremely high quality of the recombinant three finger proteins we obtained, our method provides a competitive alternative to either their native counterparts or chemically synthetic ones and should facilitate related research and applications.

**Supplementary Information:**

The online version contains supplementary material available at 10.1186/s12934-024-02316-1.

## Introduction

The snake venom is a large repertoire of digestive enzymes, toxin peptides and compounds. The three-finger neurotoxins (TFNs) are a collection of such toxin peptides used by the snake to kill the prey through binding and blocking ion channels in the neurological system. The α-neurotoxin binds to and block the muscle type nicotinic acetylcholine receptor (α1_2_β1γ(ε)δ nAChR) on the neuromuscular junction, leading to paralysis of the prey. The snake venom also contains various toxin peptides that have interesting properties. Some of them have analgesic effect, such as mambalgin-1 [1] and Hannalgesin [2], some of them can bind to receptors in the neurological system, such as MTα and κ-Bungarotoxin, which bind to Muscarinic α2B-adrenoceptor [3, 4] and α3β2 nAChR [5, 6], respectively. Although these toxin peptides may have attractive usage in biomedical research, not all toxin peptides are present in large quantities in snake venoms. κ-Bungarotoxin (κBtx), for example, only takes a very small fraction of the venom, was often contaminated by α-Bungarotoxin (αBtx), leading to inconsistent results in some of the earlier researches [7], and is commercially unavailable now, thus further restricted their study and usage.

Three-finger proteins (TFPs) are a class of proteins (peptides) including TFNs and their mammalian homologue that show similar three-finger structure as TFNs. These mammalian TFP homologues are referred to as the Ly6/uPAR family proteins, some famous players of which include Lynx [8–12], Slurp [13–19], which are proposed to be endogenous modulators of nAChR, the Pate (Prostate and Testis Expression) family [20–23], which play important roles in the capacitation of the sperm and fertilization and CD59, a GPI-anchored membrane protein that protects the cell from complement attack [24–27]. As the detailed biological function of many of these toxin-like proteins (peptides) still largely remains elusive, it is thus desirable to have a reliable production method for the scientific community.

Due to the complex intramolecular disulfide bonds system in TFPs, it is usually impossible to obtain correctly folded TFPs directly from *E. coli* and the expressed recombinant proteins are always in the form of inclusion body (I.B.), and all previous attempts included an additional refolding process [28–30]*.* Other expression systems were also attempted, such as *Pichia Patoris* [31–33]*, or Eukaryotic* expression systems [34]. While these attempts obtained recombinant three finger proteins (rTFP) and met the end claimed in the research, all these methods suffered from sophisticated post-purification cleavage of fusion tags and low yield. Until now, very few of these recombinant three-finger proteins (rTFPs) have been structurally validated by X-ray diffraction (XRD) protein crystallography studies. There were successful attempts using chemical synthesis, such as muscarinic toxin MT7 and MT1 [35], and the pain-killing toxin Mambalgin [1, 36, 37], but this method suffered from sophisticated synthesizing steps and high costs. As such, a universal, high yield production protocol capable of producing high quality rTFPs is desired.

We previously reported the production of bioactive recombinant α-Bungarotoxin (rec-αBtx) [38], in which we used radioimmunoassay to determine the optimal refolding conditions. For other TFPs, without a valid activity determination method, it is usually hard to tell which refolding condition is optimal for a particular TFP. Here we introduce an efficient, productive pipeline that we used to generate various rTFPs, most of which were structurally and biochemically validated.

## Materials and methods

**Fig. 1:**
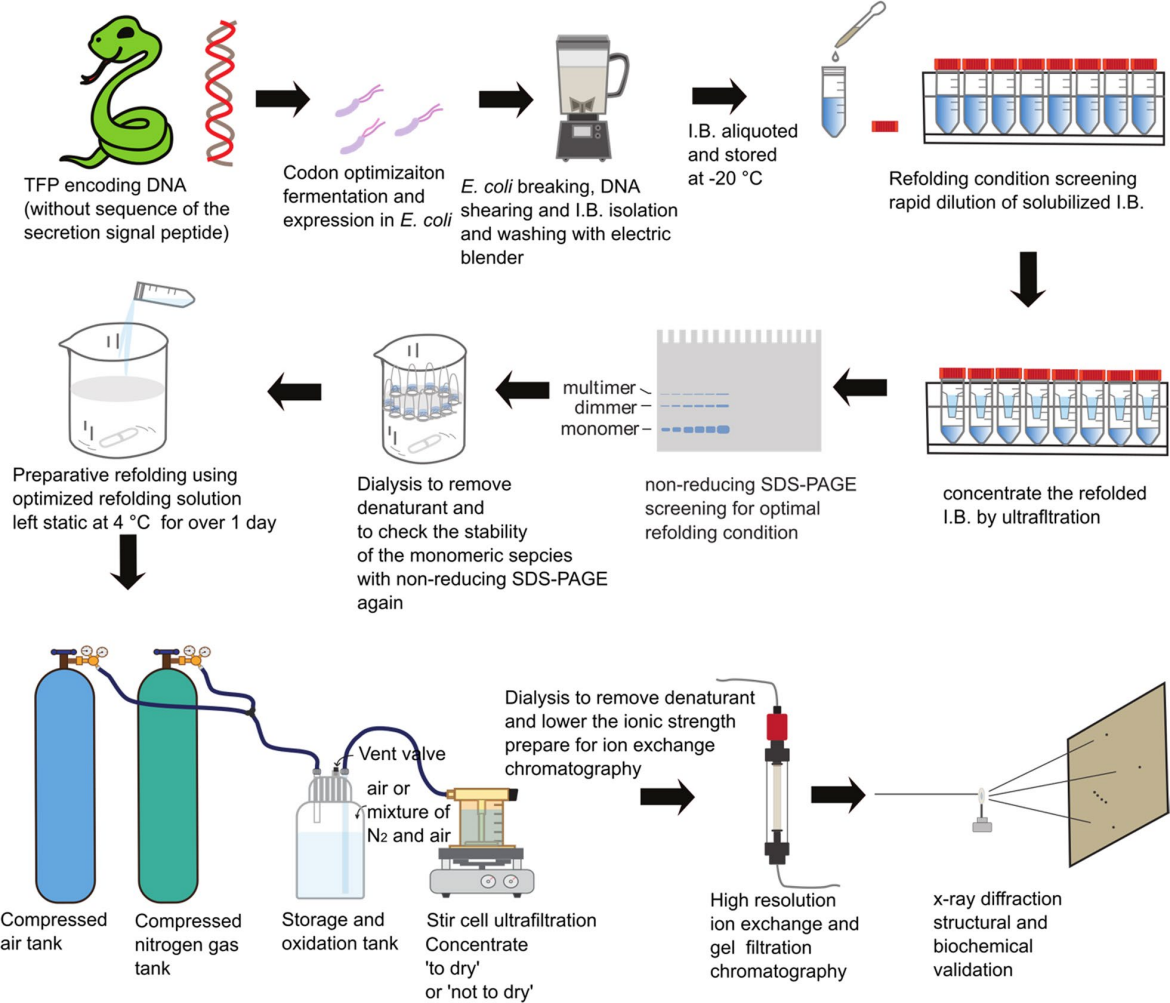
Experimental flow chart illustration

The Buffers and Mediums used are summarized in detail in Additional file 5: Table S1. Restriction enzymes were from Takara or New England Biolabs. All chemicals were from Sigma-Aldrich unless otherwise stated. The overall experimental design is illustrated in Fig. 1.

### Vector construction, *E. coli* fermentation and inclusion body extraction

Genes encoding the toxin proteins were codon-optimized to *E. coli*-preferred codons with the Jcat software [39] and synthesized (Genscript Inc, Integrated DNA Technologies Inc) with NdeI (CATATG) or NcoI (CCATGG) site as described before [38] on the 5’ end and a termination codon (TAA or TAG) at the 3’ end just before the XhoI sites (Additional file 4). The genes were inserted into the NdeI and XhoI sites of pET30b (Novagen) or the NcoI and XhoI sites of pET22b (Novagen), and the reconstructed expression vector was transformed into the *E. coli* cloning host JM109 or Stellar^™^ (Takara). Positive clone was confirmed by Sanger sequencing and then transformed into expression host BL21(DE3), and was tested for protein expression at 37 °C in 2 ml (or 5 ml) culture in 10 ml (or 20 ml) test tubes, in which IPTG was added to 1 mM at OD_600_ of 0.7, and carried on for 4 h. For analysis on SDS-PAGE gels, 1.5 ml of induced culture were pelleted down, and resuspended in 100 μl of 2xSDS-PAGE loading dye, boiled for 5 min and run on 15% SDS-PAGE gel. The protein bands of interest from the SDS-PAGE gel were digitized by measuring the pixels' value sum of each band (referred to band amount hereafter) from the inverted grey scale picture of the gel graph with Image J [40]. For rTFPs with observable expression on SDS-PAGE, *E. coli* cells were further fermented either with a home-made 5 L bioreactor or a BioFlo3000 10 L Bioreactor (New Brunswick Scientific). Both bioreactors' fermentation temperature was set to 37 °C. For BioFlo3000 10 L bioreactor, the volume of the base fermentation medium was 6 L, and the stir speed was looped to DO (dissolved oxygen), which was set to 20%, with a minimum speed set to 500 rpm and maximum speed set to 1000 rpm, air flow was set to 10 L/min. The pH value of the medium was automatically kept at around 7 with addition of concentrated ammonium hydroxide solution throughout the fermentation process. For the home-made 5 L bioreactor, the volume of the base fermentation medium was 3 L, and a fixed stirring speed of 1000 rpm was used, air flow was set to 5 L/min. The pH value of the medium was kept at around 7 with addition of concentrated ammonium hydroxide solution after the first DO peak, at 40 min interval by manually sampling the culture and measuring the pH value with pH test strips. Culture density was monitored by measuring the OD_600_ of the diluted culture (with 1xPBS), so the measured OD_600_ value was less than 0.3, at 1 h or 20 min interval, when the culture’s OD_600_ was below or above 10, respectively.

Typically, 4 ~ 10 freshly transformed *E. coli* colonies were inoculated into 250 ml/500 ml (for 5 L home-made bioreactor or 10 L BioFlo3000 bioreactor, respectively) of 2xYT medium supplemented with 0.5% glucose and 100 μg/ml ampicillin or 50 μg/ml kanamycin according to the pET plasmid used. The *E. coli* culture was incubated in a shaking incubator at 37 °C/250 rpm to an OD_600_ value of 0.7, then inoculated into the base fermentation medium and fermented using the fed-batch strategy with addition of 40% glucose to 1% each time DO started to peak, until the OD_600_ of the culture reached 19 ~ 22. IPTG was then added to 1 mM to induce protein expression for 4 h, after adding IPTG, feed medium was switched to 1/10 culture volume of GYT each time DO started to peak. The growth of *E. coli* dramatically slowed down after addition of IPTG, as could be observed by the increased time interval between each feed and decreased increment rate of OD_600_ of the culture. Normally, 160 ~ 200 or 350 ~ 400 g of bacteria pellets (wet weight) could be obtained with the 5-L home-made bioreactor or BioFlo3000 10 L bioreactor, respectively. The *E. coli* pellets were collected and stored at − 20 °C as 50 g aliquots. To obtain the inclusion bodies, 200 g of bacteria was thawed in 1 L of lysis buffer supplemented with 2 mg of chicken egg lysozyme per gram of bacteria pellets of was then added and mixed well using a bench-top electric blender (KitchenAid KSB1575ER Blender, or Philips HR2094/00 blender) at their top-speed. The mixture was incubated on ice for 1 h and sheared with the blender at top-speed for 60 s and cooled in the cold room for 15 min, the shearing process was repeated twice until the solution become less sticky, which was then centrifuged at 10,000*g*/4 °C/15 min. The supernatant was discarded, and the pellets were subjected to a new round of resuspension-shearing-centrifugation process until the pellets became compact and supernatant turned from turbid to translucent. The pellets were finally resuspended evenly in 1 ~ 2 L of lysis buffer (the exact volume depends on the total amount of crude I.B., which should be kept within 1 g/tube (wet weight) with the help of the electric blender and aliquoted to 50 ml/tube in 50 ml conical tubes, pellet down by centrifugation at 8000*g*/10 °C/15 min and stored at − 20 °C until use.

### I.B. solubilization and refolding screen

To solubilize the I.B., a solubilization buffer containing 50 mM Tris–HCl (pH 8.0), 8 M urea or 6 M guanidine-HCl and 5 mM 2-ME was used. The choice of the solubilization buffer was based on the solubilization effect and contaminating protein level. Taken αBtx for example, this toxin refolded poorly in the presence of contaminating proteins and its I.B. was solubilized well with a solubilization buffer containing 8 M urea. So, after solubilization with 50 mM Tris base, 8 M urea, and 5 mM 2-ME, and centrifuged for 28,000*g*/10 min/4 °C to get rid of insoluble bacteria debris, the pH of the supernatant was adjusted to 8.5 with concentrated HCl solution and further absorbed with Q Sepharose FF media (2 ml of solution/ml of Q media) equilibrated with 50 mM Tris–HCl (pH 8.5), 8 M urea. Attention should be paid to avoid using high concentrations of reducing chemical reagents (like 100 mM of 2-ME) at the solubilization stage, which will lead to low refolding efficiency that may be caused by blocking the formation of disulfide bonds. After absorption, the I.B. was ready for refolding. For other toxins with higher expression level and more compact inclusion bodies, a solubilization buffer containing 50 mM Tris–HCl (pH 9.0), 6 M guanidine-HCl, 5 mM 2-ME was used. A simple but useful test to assess the compactness of the I.B. is to poke the I.B. after the last washing step at the bottom of the 50 ml conical centrifuge tube with a 200 μl pipette tip, if the I.B. is compact, the pipette tip should be able to stand, and if the I.B. is loose, the pipette tip will tend to fall. This is particularly useful when selecting between urea based and guanidine hydrochloride-based solubilization buffers.

Refolding condition was optimized with a screening protocol scouting for NaCl concentration (0 or 200 mM), L-cysteine concentration (0 to 64 mM), L-arginine-HCl (0 or 0.5 M, pH 8.8), and detergent, such as NDSB-201(0 or 0.2 M), etc. Standard refolding trial was made by diluting 200 μl of I.B. solution into 10 ml of refolding screen solution with detailed recipe presented in Material and Method. After dilution, the solutions were left at 4 °C overnight, and concentrated each with Amicon Ultra-15 (Millipore, 3 kDa NMWL) ultrafiltration device to about 200 μl. The retention was centrifuged at 18,000*g*/4 °C for 15 min and the supernatant analyzed with non-reducing SDS-PAGE. Generally, mis-paired disulfide bonds could lead to formation of intermolecular disulfide bonds and multimeric species, which are shown as a ladder pattern on non-reducing SDS-PAGE (Additional file 1: Figure S1, grey arrow), while correctly paired disulfide bonds facilitate formation of monomeric species, which are usually shown as the smallest band on non-reducing SDS-PAGE (Additional file 1: Figure S1, black arrow). The rest of the concentrated solutions were each divided into three parts and dialyzed against low ionic strength buffer with various pH values, such as 20 mM sodium acetate (NaAc, pH 5.0), 20 mM HEPES (pH 7.0, adjusted with NaOH), or 20 mM Tris–HCl (pH 8.0), using a set of home-made micro-dialysis devices [41]. Finally, the dialyzed solution was centrifuged at 18,000*g*/4 °C for 15 min. The supernatant was analyzed with non-reducing SDS-PAGE to assess the stability of different refolding species (i.e., monomer and multimers). For quantitative comparison between different refolding conditions, two metrics, ‘monomer ratio’ and ‘relative monomer amount’ were calculated. ‘Monomer ratio’ is the ratio between the band amount of monomeric species and the band amount of the total, which is the band amount sum of monomeric, dimeric, trimeric, tetrameric, etc. species, if presented. ‘Relative monomer amount’ (shown as ‘rel. monomer amount’ in Additional file 1: Figure S1) was calculated by dividing the monomer band amount from each condition with the highest monomer band amount for the same rTFP in refolding screen experiment. By simply eyeballing the gel graph, or more accurately by comparing the ‘rel. monomer amount’ and ‘monomer ratio’, the best refolding condition was selected, which gave relatively high level of monomeric species shown by high ‘rel. monomer amount’ and ‘monomer ratio’, with relatively less usage of L-cysteine and without usage of L-arginine and NDSB-201. For some rTFNs, such as rec-κΒtx, which is a dimmer not interconnected with inter-chain disulfide bonds in its native form, we looked on non-reducing SDS-PAGE for conditions that produced the least amount of dimmer and highest amount of monomer, as these dimmers (which are interconnected with disulfide bonds) could be very hard to be separated from native dimeric rec-κΒtx (data not shown).

### Preparative refolding of rTFPs

For preparative refolding, freshly solubilized I.B. was poured all in once at a volume ratio of 1:50 into a freshly prepared, ice-cold refolding solution which was stirred rapidly by a magnetic bar throughout the whole process. The refolded solution was left static over one day at 4 °C and concentrated with a compressed nitrogen-gas (or air) driven ultrafiltration device (350 ml Amicon Stirred Cell, 3 kDa NMWL membrane, Millipore). As the ultrafiltration device’s volume is small compared to the total volume of the refolding solution (i.e., several liters), repeated refilling was needed where O_2_ in the air was brought in, helping cysteine molecules in the solution to gradually react to form cystine and precipitated out, during which the disulfide bonds of the rTFP also formed. Considering this, we designed and crafted a ‘storage and oxidation tank’ and connected it in serial in between the compressed gas tank and the stirred ultrafiltration cell (Fig. 1). The ‘storage and oxidation tank’ not only eliminated the needs for repeated refilling of the stir cell during the ultrafiltration, but also served as a reaction vessel, making it easier in selection of compressed nitrogen gas or air or the mixture of the two, ensuring complete and homogenous oxidation formation of disulfide bonds under higher dissolved oxygen environment with the high pressure. Close to the end of the ultrafiltration process, the refolded solution was either concentrated to a very small volume of several ml (‘not to dry’), or ‘to dry’, leaving no visible liquid on the ultrafiltration membrane, depending on the type of the rTFPs being refolded. For some toxins, like recombinant MTα (rec-MTα), Hannalgesin (rec-Hannagesin), mouse Pate B (rec-mPateB), κ-Bungarotoxin (rec-κBtx), α-Bungarotoxin (rec-αBtx), it is better to concentrate to ‘dry’, which dramatically increased the purity and quality of the final product. For other toxins we tried, such as recombinant mambalgin-1 (rec-Mambalgin-1), mouse and human Slurp1 (rec-mSlurp1 and rec-hSlurp1), concentrating to dry significantly lowered the final yield. So, trial experiments should be done at this point. The concentrated product was then re-solubilized with a low ionic strength buffer, which was pre-determined in the dialysis experiment. Normally, proteins with isoelectric point (pI) over 7 was re-solubilized in 30 to 50 ml of 20 mM NaAc (pH 5.0), while proteins with pI less than 7 was solubilized in 20 mM Tris–HCl (pH 8.0) or 20 mM HEPES (pH 7). The solution was then filtered with a 0.2 μm filter and applied to mono S 5 50 GL or mono Q 5 50 GL column driven by a FPLC system (ÄKTA™ Purifier, GE Healthcare) based on the isoelectric point (pI) of the proteins. Bound proteins were eluted with a linear gradient of NaCl to 1 M. The eluted peaks were again analyzed by non-reducing SDS-PAGE. Those eluted later usually contained contaminating proteins, or species inter-connected by intermolecular disulfide bonds. For those concentrated 'not to dry’ but to small volume, an additional dialysis step was usually added, in which the concentrated solution was dialyzed against the low-ionic strength buffer before being subjected to cation exchange chromatography. For the proteins we tried, a single, large peak was usually seen using the mono S column, and several large peaks were seen using the mono Q column, in which the target species was usually contained in the first peak. At this stage, the refolded rTFP was pure, but for crystal growth, gel filtration was usually done with a Superdex 75 10 300 GL column (GE Healthcare), to further increase the purity of the product and to buffer-exchange to 200 mM ammonium acetate (pH 7 °C).

### Native gel shift assay

5 μg of HAP peptide [42, 43] were mixed with 5 μg of each of the rTFPs, respectively (with molar ratio HAP:rTFP > 6), incubated at room temperature for 15 min, and run on a 15% native PAGE gel with 50 mM NaAc (pH 5.0) at 120 v/60 min/ 4 °C. For the binding assay with the nicotinic acetylcholine receptors, 5 μg of recombinant α-Cobratoxin (rec-αCTX), recombinant Hannalgesin ( rec-Hannalgesin), or α-Cobratoxin (αCTX) (Sigma-Aldrich, C6903) was mixed with 5 μg of the recombinant extracellular domain of the α1 subunit of muscle type nicotinic acetylcholine receptor (rec-α1ECD) [44, 45] (molar ratio, rTFP (TFP): α1ECD = 3), incubated on ice for 15 min and run on 12% native gel (standard discontinuous PAGE gel without SDS, 6% for top layer and 10% for bottom layer) with Tris–Glycine buffer (pH 8.3, without SDS) as the running buffer, at 120 v/90 min/4 °C. Gels were stained with Coomassie Brilliant Blue G-250 as described [46].

### Labeling of rec-mPate B with fluorescence dye and visualization of binding of rec-mPate B to the mouse spermatozoa

rec-mPate B was labeled with NHS-rhodamine (Thermo Scientific) according to the manufacturer's recommended protocol. Briefly, 25 μl of rec-mPate B (27.2 mg/ml in PBS, pH 7.4) at was mixed with 20 mM HEPES (pH 7), 4.13 μl of 18.9 mM NHS-Rhodamine DMSO solution and incubated at room temperature for 60 min, and dialyzed exhaustively against 20 mM HEPES, 0.15 M NaCl. Mouse spermatozoa was obtained as described [47], and was mixed with 1:1000 dilution of the Rhodamine labeled rec-mPate B, washed three times with PBS, and observed under a laser confocal fluorescence microscope.

### X-ray protein crystal diffraction structural validation of rTFPs

For crystallization of rec-αBtx, rec-αBtx was mixed with HAP peptide [42] at a molar ratio of 1:1.5, incubated at room temperature for 30 min and then diluted 100 fold with 20 mM NaAc, pH 5.0 and applied to mono S column. Bond protein was eluted with linear gradient of NaCl to 1 M and the sharp peak containing the rec-αBtx-HAP complex was collected, pooled, and concentrated to about 13 mg/ml, dialyzed against 0.1 M HEPES (pH 7.0) exhaustively at 4 °C. For other rTFPs, purified toxin proteins were concentrated to 80 to 150 mg/ml with Amicon Ultra-15 and Amicon Ultra-0.5 (3 kDa NMWL) tubes. Sitting drop crystal screening was done using a robotic system (Crystal Gryphon, Art Robbins Instrument). Hanging drop method was then done manually to optimize the growth condition, by mixing equal volume of well solution and the toxin protein and incubating both at 4 °C and 18 °C. Crystals were then harvested and stored in cryo-conditions and X-ray diffraction data of rec-kBtx, rec-mambalgin 1 and rec-αBtx-HAP complex were collected either with a Rigaku MicroMaxTM-007 home X-ray source coupled with an R-AXIS IV +  + image plate. For rec-MTα crystals, X-ray diffraction data was collected at Advanced Photon Source (Argonne National Laboratory, Lemont, IL). The X-ray diffraction data of rec-Hannalgesin and rec-αCTX were collected at Advanced Light Source (Lawrence Berkeley National Laboratory, Berkeley, CA). Data was processed with HKL2000 [48] or IMosflm [49], Pointless, Aimless [50], Ctruncate from the CCP4 suite [51], Molecular Replacement, structure build and refinement was done in Phenix [52] and Coot [52]. Structural visualization, alignment, and calculation were done in open source PYMOL (Version 2.0 Schrödinger, LLC).

## Results

### Our pipeline is applicable to a wide variety of TFPs with high yield, with 6 rTFPs being structurally validated.

**Fig. 2:**
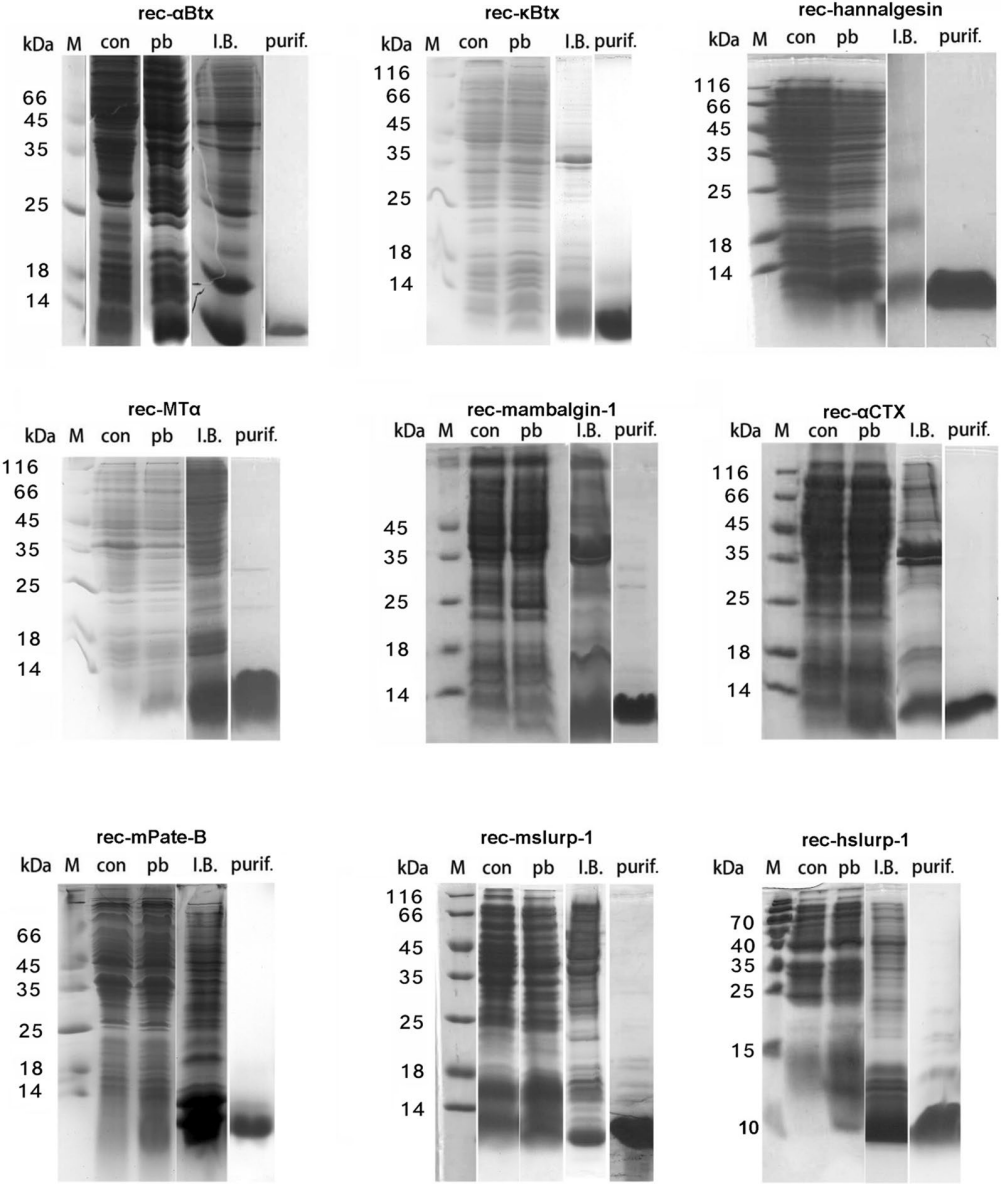
**SDS-PAGE analysis of rTFPs at different stages of production**. *con*: control (not induced E. coli cells), *pb* IPTG induced E. coli cells, *I.B.* isolated inclusion bodies, *purif.*: purified final product (in non-reducing SDS-PAGE)

Our idea is to use *E. coli* as the workhorse to produce high quality TFPs of biomedical interests. Our pipeline includes codon optimization of the encoding DNA sequence for *E. coli* expression, recombinant protein expression in *E. coli*, isolation of I.B., refolding condition screening, preparative oxidation refolding and purification, structural validation with x-ray diffraction and biochemical methods (Fig. 1). From construction of the expression vector with known protein or encoding DNA sequences, production of a purified rTFP usually took 4 ~ 5 weeks. For each of the rTFPs, non-reducing SDS-PAGE were carried out to visualize the monomeric and multimeric species with inter-molecular disulfide bonds and to check the purity of the final product (Fig. 2). For a couple of TFPs of various origin (Additional file 6: Table S2), our pipeline was shown to be robust and successful (Fig. 2, Additional file 7: Table S3, Additional file 4: Materials).

**Fig. 3:**
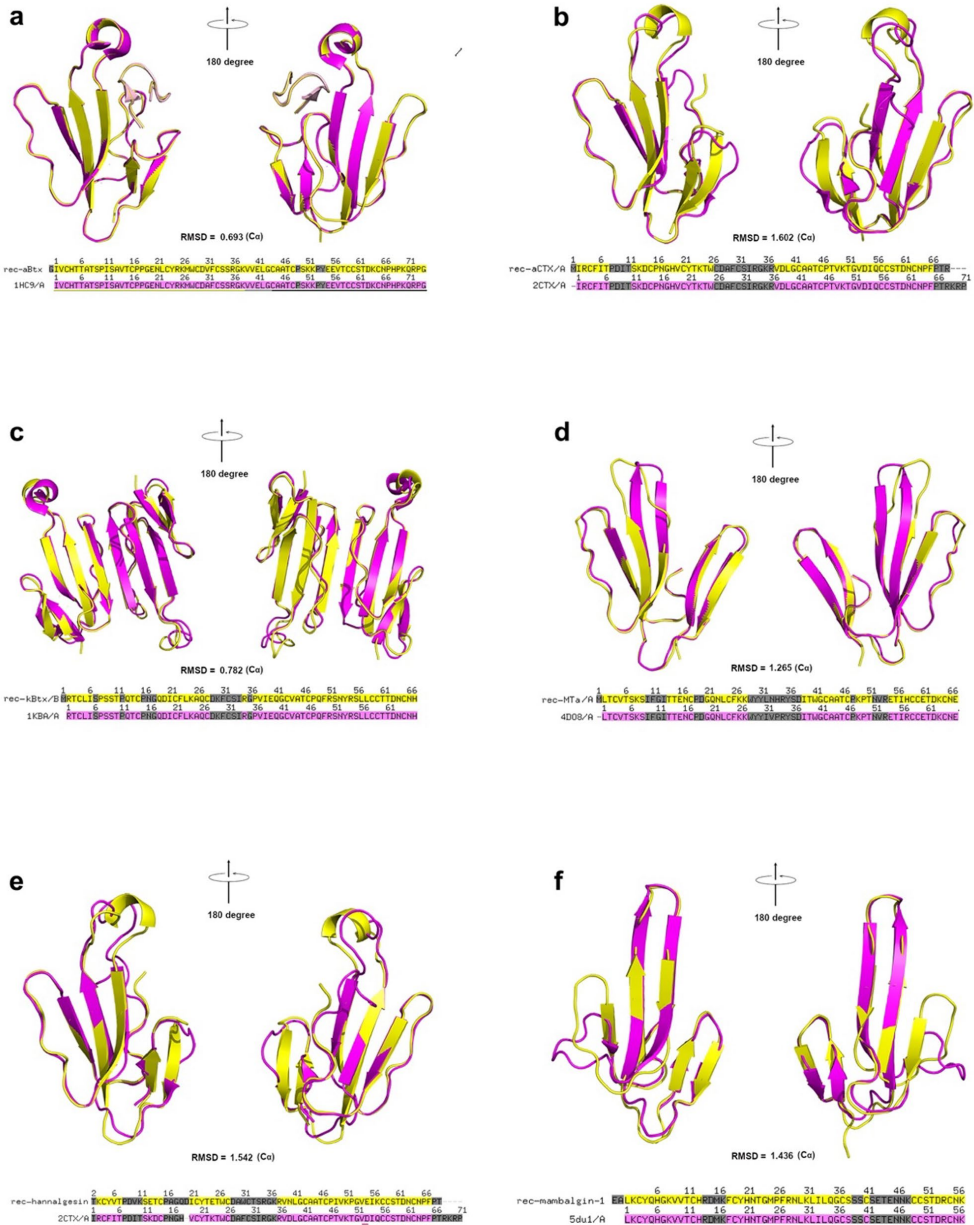
Structural alignment of the crystal structure of rTFPs and their natural counterpart or most homologous natural counterpart. Yellow: rTFP, Magenta: Reported native (homologous) or synthetic counterpart. **a.** rec-αBtx-HAP vs αBtx-HAP; **b.** rec-αCTX vs αCTX; **c.** rec-κBtx vs κBtx; **d.** rec-MTα vs MT1; **e.** rec-Hannalgesin vs αCTX; **f.** rec-Mambalgin-1 vs Mambalgin-1. RMSD was calculated based on the coordinates of C_α_ of the aligned structures

To structurally validate the rTFPs, we screened for crystal growth for each rTFP we purified (the conditions for crystal growth are listed in Additional file 4: Materials). Most of our rTFPs’ crystals were formed at high protein concentrations (Additional file 4: Materials). They were beautiful looking under the microscope with polarized light (Additional file 2 Figure S2), and diffracted x-ray quite well. Six rTFPs’ structures were solved with x-ray crystal diffraction data using molecular replacement with known homologous structures. The statistics for the diffraction data were summarized in Table 1. From the structural alignment of the solved structures with their native counterparts (such as rec-aBtx-HAP complex, rec-αCTX, rec-kBtx, rec-mambalgin) or with their most homologous native counterparts (such as rec-Hannalgesin and rec-MTα, whose crystal structures were not reported, known homologous TFPs with known structure, such as αCTX and MT1, respectively, were used as the alignment counterpart). Our rTFPs are shown to be almost identical to their natural counterparts, with one or several additional amino acids at the N-terminal (a.a. sequences are shown in Additional file 4: Materials and the additional a.a. sequences are highlighted in red color), which is a unique mark for their recombinant origin) (Fig. 3).

**Table 1:** Data collection and refinement statistics

		rec-Mambalgin-1	rec-κBtx	rec-αBtx-HAP	rec-Hannalgesin	rec-MTα	rec-αCTX
Resolution range (Å)		24.09–2.49 (2.579–2.49)	36.71–1.8 (1.864–1.8)	33.35–2.4 (2.486–2.4)	39.46–2.2 (2.279–2.2)	29.28–1.8 (1.865–1.8)	30.77–1.57 (1.626–1.57)
Space group		P 1 21 1	P 1	P 1 21 1	P 32	P 63	P 3 2 1
Unit cell	Length (Å)	30.984 80.753 54.032	27.0899 30.9899 39.43	42.5295 73.1098 79.8397	65.5095 65.5095 164.752	58.555 58.555 35.395	73.9695 73.9695 110.841
	Angle (°)	90 90.47 90	70.44 75.97 71.94	90 103.68 90	90 90 120	90 90 120	90 90 120
Multiplicity		3.9 (4.0)	4.0 (4.0)	7.6 (7.7)	6.1 (6.0)	9.3(7.2)	21.7 (20.6)
Completeness (%)		94.97 (93.12)	93.40 (89.76)	96.32 (94.14)	99.76 (99.58)	99.88 (99.84)	99.83 (99.79)
Mean I/sigma (I)		20.12 (7.47)	16.92 (8.91)	10.58 (4.53)	11.67 (3.38)	53.4 (17.65)	21.76 (3.96)
Wilson B-factor		55.19	13.48	18	41.94	21.26	20.76
R-merge		0.04624 (0.1469)	0.05006 (0.1079)	0.1477 (0.4164)	0.08822 (0.5762)	0.049(0.129)	0.08866 (0.8743)
R-meas		0.05376 (0.1683)	0.05787 (0.1247)	0.1584 (0.4462)	0.09657 (0.631)	0.052(0.137)	0.09088 (0.8962)
CC1/2		0.997 (0.978)	0.998 (0.985)	0.992 (0.943)	0.996 (0.904)		0.999 (0.951)
R-work		0.2179(0.3432)	0.1658 (0.2067)	0.2393 (0.2844)	0.2115 (0.2708)	0.1897 (0.3007)	0.1736 (0.2363)
R-free		0.2670 (0.3537)	0.1912 (0.2233)	0.2750 (0.3626)	0.2431 (0.3349)	0.2202 (0.3696)	0.1844 (0.2515)
macromolecules		1845	1018	2671	6094	532	2040
ligands		15	0	96	25	28	39
RMS (bonds)		0.008	0.007	0.006	0.007	0.009	0.012
RMS (angles)		1.18	1.01	1.09	0.98	1	1.25
Average B-factor		72.89	20.55	23.99	63.69	28.06	26.68
macromolecules		73.10	19.43	23.52	63.95	26.95	24.44
ligands		68.76		63.94	72.89	47.62	51.78

(Values in parentheses are for the highest-resolution shell)

Though it was generally considered very difficult to obtain rTFPs using either *E. coli* or other expression systems, with our pipeline, we could repeatedly obtain over one hundred mg of rec-MTα, rec-Hannalgesin, rec-αCTX and rec-mPate B, tens of milligrams of rec-mambalgin-1, Slurp1 and milligrams of rec-kBtx and rec-αBtx (Additional file 4: materials) through one round of experiment (usually finished within 4 ~ 5 weeks). The detailed expression level, final yield and refolding species analysis are summarized in Additional file 7: Table S3.

### Most useful scouting conditions for refolding rTFPs are cysteine, salt concentration, and pH

The optimized refolding condition for each recombinant neurotoxin was summarized in the Additional file 4: Materials. The most critical factors are the concentration of sodium chloride and L-cysteine, and pH value. A weak basic solution, with different NaCl and L-cysteine concentration provided the essential refolding solution. Without Tris base, increasing L-cysteine concentration significantly alter the pH from neutral to acidic, leading to lowered yield of monomeric species (data not shown). L-arginine [53–56] and NDSB-201 [57, 58], two known supplements which are widely used in inclusion body refolding, even though significantly increased the yield of monomeric species in the screening experiment as reflected by non-reducing SDS-PAGE (Additional file 1: Figure S1), lead to formation of a lot of precipitates in the subsequent dialysis removal of these supplements (data not shown), and thus didn’t help much. What’s more, L-arginine and NDSB-201 are relatively expensive and not cost-effective in large scale production. Taken together, L-arginine and NDSB-201 are generally not recommended for refolding rTFPs, at least for those we tried. We generally only visually check the gel graph to determine the refolding condition for the scaled-up production, according to the band intensities of the monomer. For more accurate estimation, protein band corresponding to different refolding species was quantified with densitometry measurement and two metrics, ‘monomer ratio’ and ‘relative monomer amount’ were calculated, which would be helpful for selection of the optimal refolding condition. The condition selected by eyeballing the gel gave good if not best ‘monomer ratio’, ‘relative monomer amount’ values (Additional file 1: Figure S1).

Normally, rTFPs with high isoelectric point (pI) remained soluble upon challenge with weak acidic solution (such as 20 mM NaAc, pH 5.0), while certain mammalian three finger toxin-like protein, such as Slurp1, remained soluble only in neutral and slight basic solutions, such as 20 mM HEPES (pH 7.0) and 20 mM Tris–HCl (pH 8.0).

### Complete oxidation is the key to the production of high quality rTFPs

It is common to see I.B. refolding protocols in which people dissolve the I.B. with solutions containing high concentration of reducing agents (such as 100 mM β-mercaptoethanol or 2-ME). While these agents are useful in keeping the free cysteine residue in reduced form and it might not be a problem in certain cases, we found 100 mM 2-ME in I.B. solubilization buffers inevitably lead to failed refolding experiments, which was shown by the extremely low yield and formation of multimeric species [38], thus should be avoided when solubilizing the I.B. For correct disulfide bonds pairing between the cysteine residues, a classical and widely used approach is the disulfide shuffling or mixed disulfide bond reactions, in which a predefined redox pairs such as a fixed ratio of reduced-glutathione: oxidized-glutathione, or cysteine:cystine are used [59–61]. In our pipeline, we used a simple, straightforward approach by scouting cysteine and NaCl concentration in screening of refolding conditions, and we noticed that different rTFPs had different sensitivity to cysteine concentration in the refolding experiment (Additional file 1: Figure S1). We would choose a condition with the less usage of L-cysteine that produced the highest level of monomeric species, as shown by non-reducing SDS-PAGE. In preparative refolding, we used compressed N_2_ gas and/or air to drive the ultrafiltration device (Fig. 1). Because of the large volume of the diluted refolding mixture, we had to frequently reopen and refill the device, which inevitably brought air in. The O_2_ in the air should help the oxidization of cysteine to form cystine, and disulfide bonds in the rTFPs. In refolding of rec-kBtx, we found N_2_ gas was not as good as compressed air, which dramatically decreased the multimeric species in the final product (data not shown), and only the purified rec-kBtx from this special protocol yielded crystals. Clearly, ultrafiltration with the stirred cell is not only a physical process, but also a biochemical process in which the dissolved oxygen level is critical for the correct and complete formation of disulfide bonds. Considering this, we designed and crafted a 'storage and oxidation tank' (Fig. 1), which acted as an oxidation reservoir for the refolded protein mixture and gas, eliminating the needs for opening the ultrafiltration cell to refill and making it easier to adjust the ratio between air and N_2_.

**Fig. 4:**
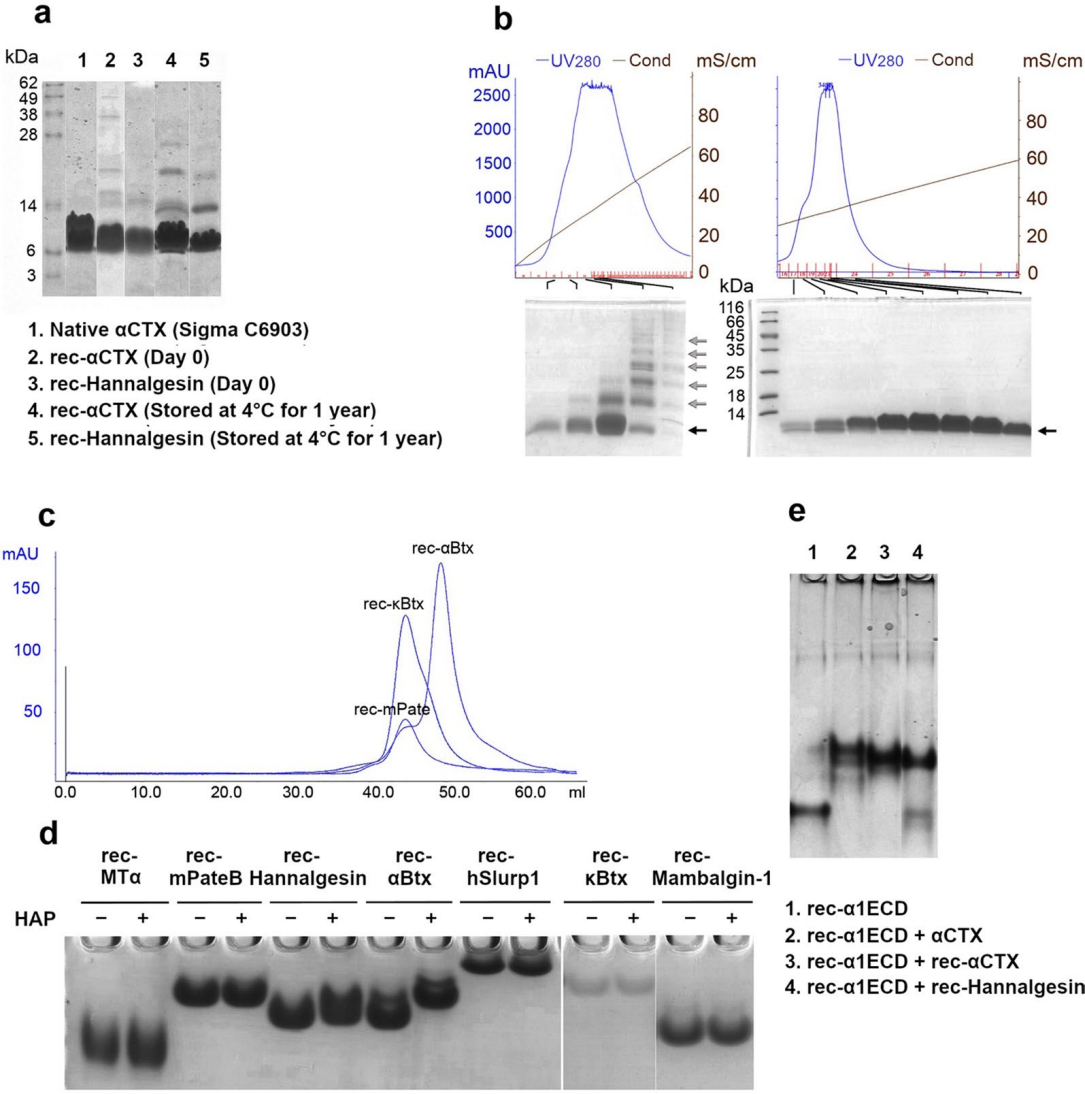
Biochemical characterization of the rTFPs **a**. Stability of rec-αCTX and rec-Hannalgesin upon prolonged storage at 4 °C, analyzed with non-reducing SDS-PAGE; **b**. ‘Concentrate to dry’ strategy (right, with black arrow pointing to the monomeric species) efficiently removed multimeric species that were hard to be separated with the mono S column (left, in which the refolded product was not ‘concentrate to dry’, grey arrow); **c**. Gel filtration analysis of rec-αBtx, rec-mPate B and rec-κBtx. **d.** Native gel shift assay of various rTFPs with HAP peptide. **e**. Native gel shift assay of rec-α1ECD with native αCTX, rec-αCTX and rec-Hannalgesin

A typical preparative ultrafiltration procedure took about 4 weeks, during the last a few days of which a large amount of white precipitate (which turned out to be cystine, the oxidization product of cysteine) showed up in the concentrated solution, which were found to be a sign of complete oxidation, since most of our high quality rTFPs were produced in this way. Some of our rTFPs were tested for their stability with prolonged storage at 4 °C, and were shown to be stable even after one year of storage at 4 °C, and only trace amounts of dimeric and multimeric species were found (Fig. 4a), consistent to previous report about the stability of natural TFNs [62], and proving the high quality of the rTFP from our pipeline.

### Concentrate to dry is a simple and efficient step for removing incorrectly folded species

It is interesting to note this point, since we found that multimeric species, which were generally regarded as incorrectly folded product with wrong pairing of disulfide bonds, were inevitable as a part of refolding product and hard to be completely separated from the correctly folded species using common chromatography approaches, such as gel filtration (data not shown) and ion exchange. However, it turned out that ultrafiltration of the refolded product to dry dramatically increased the purity for some rTFPs (Fig. 4b). It is possible that incorrectly folded but soluble rTFPs tend to form insoluble aggregates only when they are concentrated to extremely high concentrations, which was achieved in the ‘‘concentrate to dry’’ approach. It is thus noteworthy to try two ultrafiltration strategies, “to dry, or not to dry”, which in most cases could make a great difference.

### Recombinant rTFPs showed expected elution profile in gel filtration chromatography and were shown to be active in biochemical and morphological assays

We compared the elution volume of rec-αBtx, rec-κBtx, rec-mPate B in gel filtration column (Superdex 75 10 300 GL, GE Healthcare), and found that rec-αBtx, which were known as a monomer, eluted much later than rec-κBtx, suggesting rec-κBtx was not a monomer, which is in accordance with earlier reports [6] that κBtx exists in dimeric form and also with the solved crystal structures (Fig. 3c), while rec-mPate B elute at similar volume as rec-κBtx, suggesting rec-mPate B was also a dimmer (Fig. 4c). To test the binding specificities of the rTFPs, HAP peptide, a known peptide derived from the nicotinic acetylcholine receptor [42], was mixed with various rTFPs and separated on a 15% native PAGE gel at pH 5.0. HAP peptide was only able to shift rec-αBtx and only slightly shift rec-Hannalgesin, but not rec-MTα, rec-mPate B, rec-κBtx, rec-Mambalgin-1 and rec-hSlurp1 (Fig. 4d). Also, rec-αCTX and rec-Hannalgesin was shown to bind the extracellular domain of α1 subunit of the nicotinic acetylcholine receptor (rec-α1ECD) [45], like the native αCTX isolated from Naja Kaouthia (Fig. 4e).

To test the binding activity of rec-mPate B to sperm, we labeled rec-mPate B with NHS-rhodamine and visualized the binding of rec-mPate B to spermatozoa freshly isolated from the epididymis of the mouse under the fluorescence microscope. The preliminary result suggested binding of rec-mPate B to the head and tail of mouse spermatozoa (Additional file 3: Figure S3).

## Discussion

TFPs are a large collection of proteins with important functions and applications. Traditionally, such proteins were isolated from the venom of the snakes, with very few recombinantly obtained in the lab with in-depth analysis and verification. Because of their scarcity, unique properties and applications, these proteins are very expensive (at the level of hundreds to thousands of US dollars per milligrams) and some are not commercially available. κBtx, for example, a unique α_3_β_2_ nicotinic acetylcholine receptor binder, is not commercially available (personal communications). Because TFPs usually contain 4 to 5 pairs of disulfide bonds, it is usually very hard to recombinantly express them, and those commercially available are mostly purified from snake venoms. Some researchers used chemical synthesis that successfully obtained these rTFPs, such as mambalgin-1 and mambalgin-2 [1, 36, 37, 63–65]. However, due to the high cost in chemical synthesis and limited yields, these successful attempts did not change the overall scenario for production of other TFPs.

With our pipeline, however, milligrams to hundreds of milligrams of rTFPs could be obtained in the lab within weeks. Through extensive biochemical assays and structural analysis, we showed that our rTFPs were almost identical to their native counterparts. Since several of our rTFPs reached milligrams to hundreds of milligrams on a single lab-scale production cycle, these rTFP could thus replace their natural counterparts, and the pipeline is worth to be exploited for production of other TFPs further, which is of general interest in the field.

### Supplementary Information

**Additional file 1: Figure S1.** Refolding condition screening of rTNFs.

**Additional file 2: Figure S2.** Microscopic view of protein crystals from various rTFPs.

**Additional file 3: Figure S3.** Fluorescence microscopic picture showing the binding of rec-mPate B to the spermatozoa from mouse epididymis.

**Additional file 4: **Coding DNA sequences, a.a. sequences, protein properties, refolding conditions and key points and crystallization conditions for rTFPs.

**Additional file 5: Table S1.** Solutions and media table.

**Additional file 6: Table S2.** Properties and references of various TFPs we successfully refolded and purified.

**Additional file 7: Table S3.** Expression level, monomer fraction and relative amount from dilution refolding and final yield table for rTFPs.

## Data Availability

Coordinates and structure factors have been deposited to the Protein Data Bank with accession number of 7ULB (rec-Mambalgin-1), 7ULR (rec-kBtx), 8VY8 (rec-αBtx-HAP complex), 7ULQ (rec-Hannalgesin), 7ULS (rec-MTα) and 7ULG (rec-αCTX).
