# Supplementary figures and images for: Scalable production of recombinant three-finger proteins: from inclusion bodies to high quality molecular probes
Source: Microb Cell Fact. 2024 Feb 12;23:48. doi: 10.1186/s12934-024-02316-1 (PMC10860255; doi:10.1186/s12934-024-02316-1)

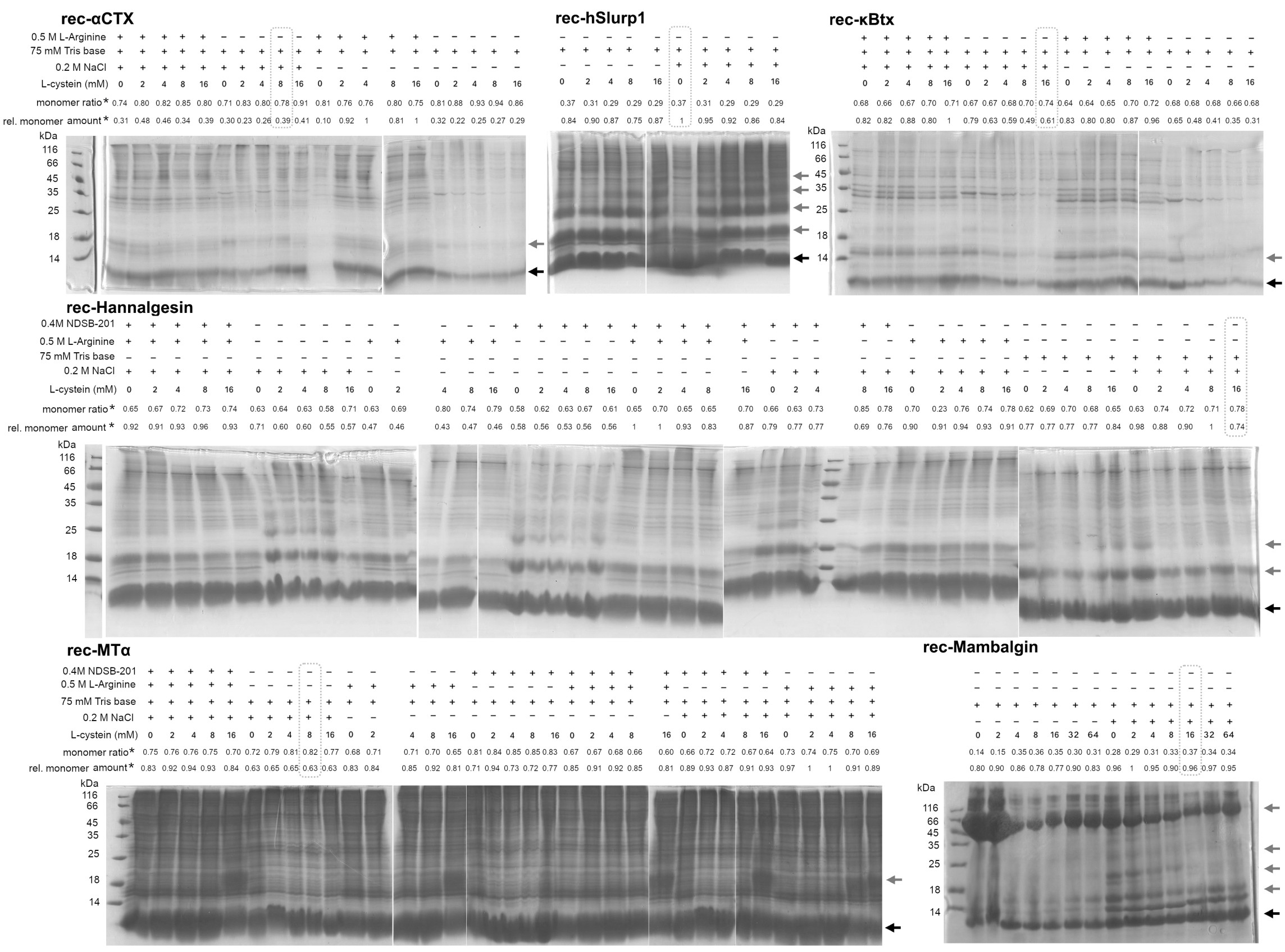

Supplement: Supplementary file 1 — Additional file 1: Figure S1. Refolding condition screening of rTNFs. [file 12934_2024_2316_MOESM1_ESM.jpg]

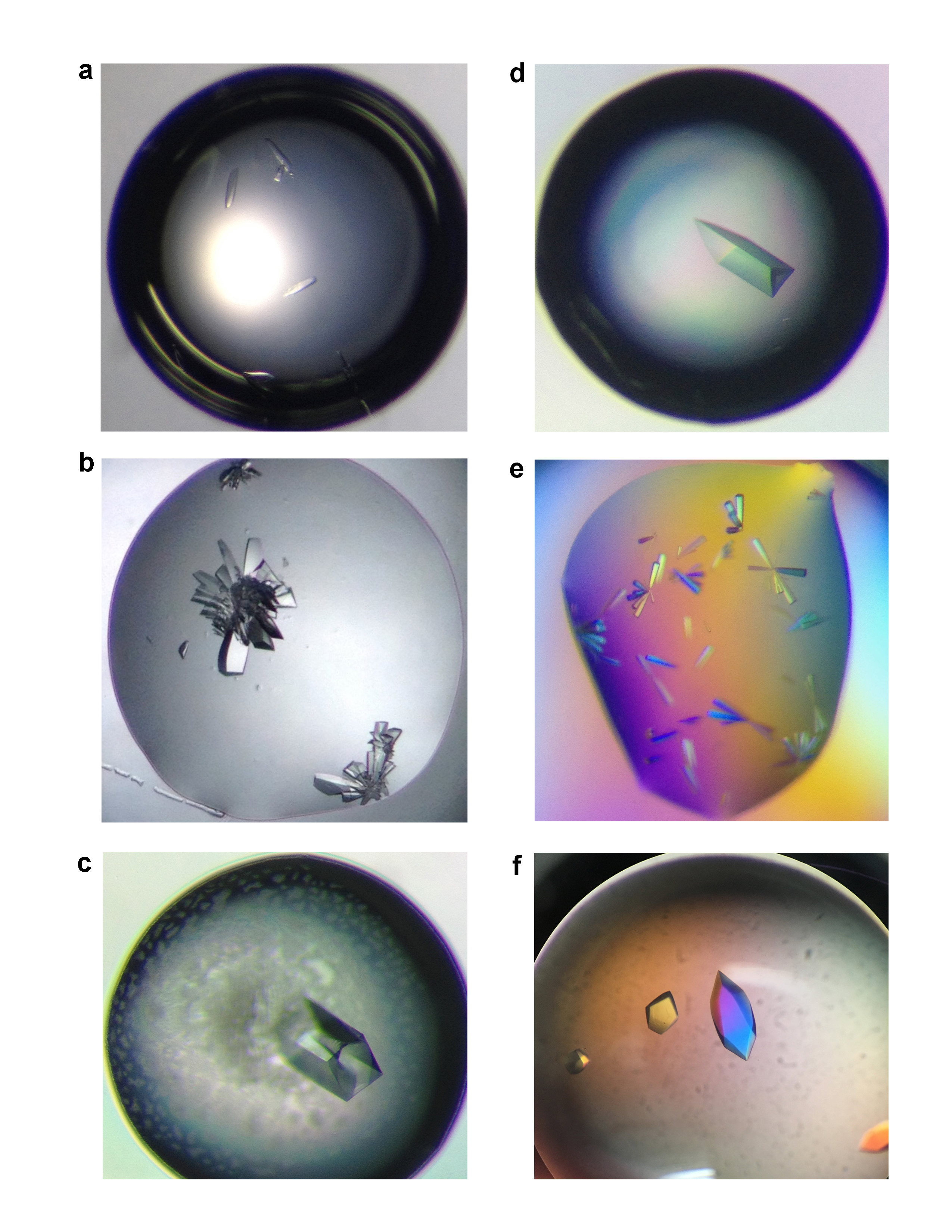

Supplement: Supplementary file 2 — Additional file 2: Figure S2. Microscopic view of protein crystals from various rTFPs. [file 12934_2024_2316_MOESM2_ESM.jpg]

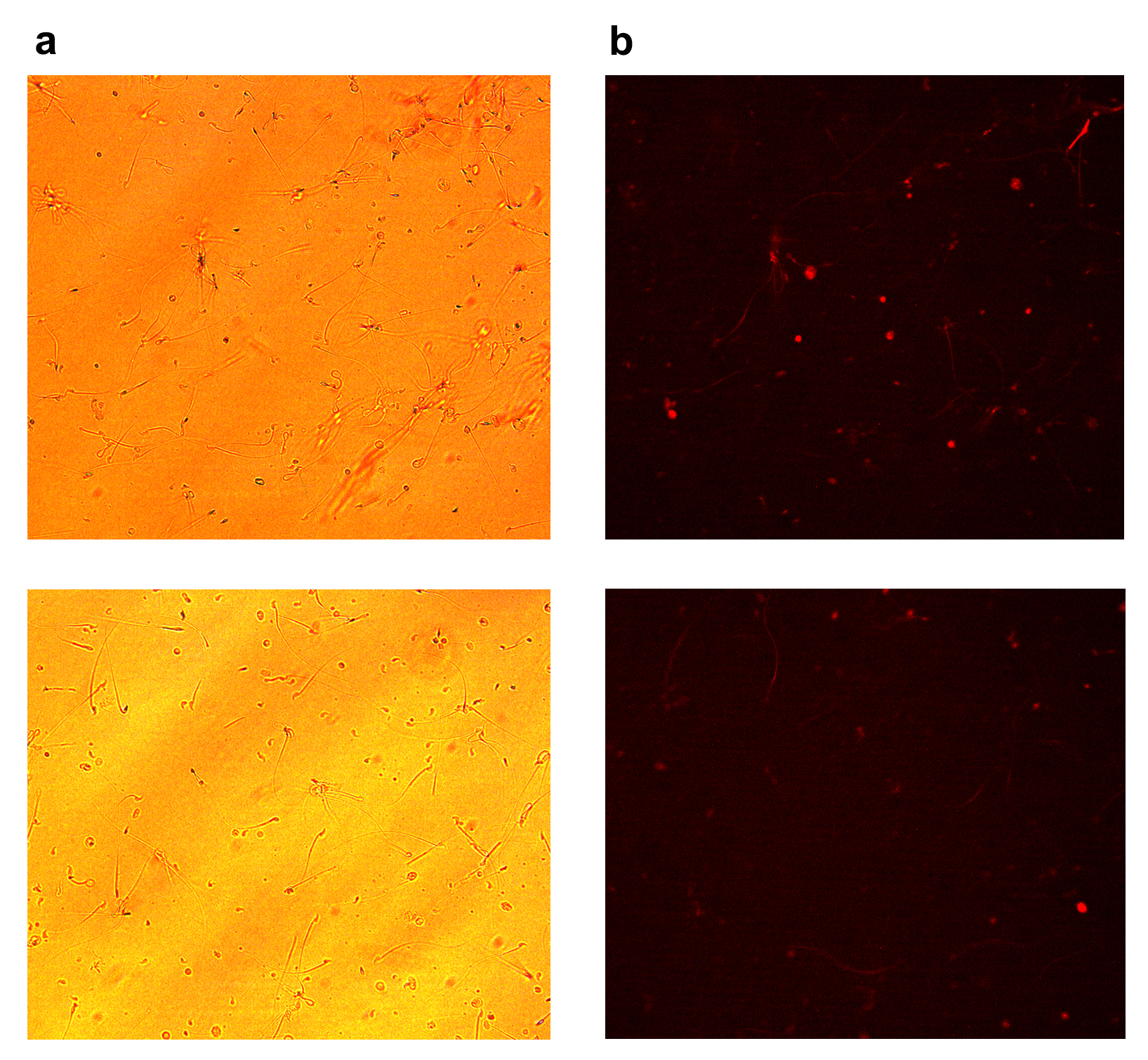

Supplement: Supplementary file 3 — Additional file 3: Figure S3. Fluorescence microscopic picture showing the binding of rec-mPate B to the spermatozoa from mouse epididymis. [file 12934_2024_2316_MOESM3_ESM.jpg]
