# Supplementary material for: Scalable production of recombinant three-finger proteins: from inclusion bodies to high quality molecular probes
Source: Microb Cell Fact. 2024 Feb 12;23:48. doi: 10.1186/s12934-024-02316-1 (PMC10860255; doi:10.1186/s12934-024-02316-1)
Supplement: Supplementary file 4 — Additional file 4: Coding DNA sequences, a.a. sequences, protein properties, refolding conditions and key points and crystallization conditions for rTFPs. [file 12934_2024_2316_MOESM4_ESM.docx]

**Additional file 5: Table 1.  Solutions and media table**

**Additional file 6 Table 2.  Properties and references of various TFPs we successfully refolded and purified**

**Additional file 7: Table 3.  Expression level, monomer fraction and relative amount from dilution refolding and final yield table for rTFPs**

**Additional file 1: Figure S1. Refolding condition screening of rTNFs.**Non-reducing SDS-PAGE analysis of refolding products from various conditions were concentrated and analyzed by 15% non-reducing SDS-PAGE (black arrow points to the monomeric species and grey arrows point to the multimeric species). Conditions selected for scaled-up production of each rTFP are enclosed with grey dashed line boxes.

* See Methods

**Additional file 2 Figure S2.** **Microscopic view of protein crystals from various rTFPs. a.** rec-αΒtx-HAP complex; **b.** rec-κBtx; **c.** rec-Mambalgin-1; **d.** rec-Hannalgesin; **e.** rec-MTα; **f.** rec-αCTX

**Additional file 3 Figure S3.** **Fluorescence microscopic picture showing the binding of rec-mPate B to the spermatozoa from mouse epididymis.** Bright field microscopic picture of mouse spermatozoa (**a**) and fluorescent graph of possible binding of rhodamine-labeled rec-mPate B to the head and tail of the spermatozoa (**b**) under the same view.

**Coding DNA sequences, a.a. sequences, protein properties, refolding conditions and key points and crystallization conditions for rTFPs**

**rec-αBtx (V31)**

**Coding sequence**:

atgggtATTGTCTGTCACACTACGGCAACGAGTCCGATCAGCGCAGTTACGTGCCCGCCGGGTGAAAACCTGTGTTATCGTAAAATGTGGTGCGATGTGTTTTGTAGCTCTCGCGGTAAAGTGGTTGAACTGGGTTGCGCAGCAACCTGTCCGAGCAAAAAACCGTACGAAGAAGTTACCTGCTGTTCTACGGATAAATGTAATCCGCATCCGAAACAGCGTCCGGGTTAA

**Translated protein sequence**:

**MG**IVCHTTATSPISAVTCPPGENLCYRKMWCDVFCSSRGKVVELGCAATCPSKKPYEEVTCCSTDKCNPHPKQRPG

**Protein parameter:** Number of amino acids: 76; Molecular weight: 8210.54; Theoretical pI: 8.36

**Refolding result:** good

**I.B. solubilization solution**: 75 mM Tris-HCl, pH 8.8, 8 M urea, 10 mM 2-ME

**Refolding solution**: 50 mM Tris-HCl, 16 mM l-cysteine, 0.2 M NaCl.

**Key process**: 1. Purify I.B. with Q Sepharose FF before refolding, and buffer exchanged to 50 mM Tris-HCl, pH 8.8, 8 M urea before refolding. 2. Ultrafiltrate to dry.

**Yield:** 0.05 mg/ g bacteria (wet pellet)

**Crystalization condtion:** mono S 5/50GL column purified rec-αBtx-HAP complex, buffer exchanged by dialysis to 0.1 M HEPES(pH 7.5), OD_280_= 11.04. 1:1 with 0.1 M HEPES (pH 7.5), 35% PEG3350, 0.2 M MgCl_2_. 18°C

**X-ray diffraction data collection:** yes

**Structural solution:** yes

**rec-αCTX**

**Coding sequence**:

atgATCCGTTGCTTCATCACCCCGGACATCACCTCTAAAGACTGCCCGAATGGCCACGTCTGCTACACGAAAACCTGGTGCGACGCTTTCTGCTCTATCCGTGGTAAACGTGTTGACCTGGGTTGCGCTGCTACCTGCCCGACCGTTAAAACCGGTGTTGACATCCAGTGCTGCTCTACCGACAACTGCAACCCGTTCCCGACCCGTAAACGTCCGTAA

**Translated protein sequence**:

**M**IRCFITPDITSKDCPNGHVCYTKTWCDAFCSIRGKRVDLGCAATCPTVKTGVDIQCCSTDNCNPFPTRKRP

**Protein parameter:** number of amino acids: 72; Molecular weight: 7962.2; Theoretical pI: 8.59

**Refolding result:** good

**I.B. solubilization solution**: 50 mM Tris-HCl, pH 8.8, 6 M Guanidine-HCl, 5 mM 2-ME.

**Refolding solution**: 75 mM Tris base, 8 mM cysteine, 0.2 M NaCl.

**key process**: Ultrafiltrate to dry.

**Yield:** 1 mg/ g bacteria (wet pellet)

**Crystallization condition**: 110 mg/ml in 200 mM NH_4_Ac (pH 7.0/25°C), 1:1 with 0.1 M HEPES (pH 7.9), 30% Jeffamine M-600 (pH 7.0), 18°C

**X-ray diffraction data collection:** yes

**Structural solution:** yes

**rec-κBtx:**

**coding sequence**:

atgCGTACCTGTCTGATTAGCCCGTCCAGCACCCCGCAAACCTGTCCGAATGGTCAAGATATTTGTTTTCTGAAGGCCCAGTGTGATAAATTTTGCAGCATTCGTGGCCCGGTGATCGAACAGGGTTGCGTTGCGACCTGTCCGCAATTTCGCTCTAACTACCGTTCACTGCTGTGCTGTACCACCGACAACTGTAATCATTAA

**Translated protein sequence**:

**M**RTCLISPSSTPQTCPNGQDICFLKAQCDKFCSIRGPVIEQGCVATCPQFRSNYRSLLCCTTDNCNH

**Number of amino acids**: 67; Molecular weight: 7406.5; Theoretical pI: 8.07

**Refolding Result:** good

**I.B. solubilization solution**: 50 mM Tris-HCl, pH 8.8, 8 M urea, 10 mM 2-ME

**Refolding solution**: 75 mM Tris Base, 16 mM cysteine, 0.2 M NaCl.

**Key process**:

1. Use compressed air to drive the Amicon Stirred Cell (200 ml).

2. Freezing and thawing the inclusion body solution (in 50 mM Tris-HCl, pH 8.8, 8 M urea, 10 mM 2-ME) and centrifugation to get rid of contaminating protein before refolding.

3. Ultrafiltrate to dry.

**Yield:** 0.1 mg purified product / g bacteria (wet pellet)

**Crystalization condtion:**117 mg/ml in 200 mM NH_4_Ac, pH 7.0, 1:1 with 0.15 M DL-malic acid pH 7.0, 20% PEG 3350 at 4 °C

**X-ray diffraction data collection:** yes

**Structural solution:** yes

**rec-MTα**

**Coding sequence**:

atgCTGACCTGCGTTACCTCCAAATCTATCTTCGGCATCACGACGGAAAACTGCCCGGACGGCCAGAACCTGTGCTTCAAAAAGTGGTATTATCTGAACCATCGTTACAGCGATATTACGTGGGGTTGCGCAGCAACCTGTCCGAAACCGACGAACGTGCGCGAAACCATCCACTGCTGTGAAACCGACAAGTGCAATGAATAA

**Translated protein sequence**:

**M**LTCVTSKSIFGITTENCPDGQNLCFKKWYYLNHRYSDITWGCAATCPKPTNVRETIHCCETDKCNE

Number of amino acids: 67 Molecular weight: 7684.7 Theoretical pI: 6.68.

**I.B. solubilization solution**: 50 mM Tris-HCl, pH 8.8, 6 M Guanidine-HCl, 5 mM 2-ME

**Refolding result:** good

**refolding solution**: 75 mM Tris base, 8 mM cysteine, 0.2 M NaCl.

**key process**: No.

**Yield:** 1~ 2 mg purified product / g bacteria (wet pellet)

**Crystallization condition**: 128 mg/ml in 200 mM NH_4_Ac (pH 7.0), 1:1(v/v) with 1.26 M sodium phosphate monobasic monohydrate, 0.14 M potassium phosphate, pH 5.6 at 18 °C

**X-ray diffraction data collection:** yes

**Structural solution:** yes

**rec-Mambalgin-1**

**coding sequence**:

atgAAGAGAGAAGCTGAAGCCTTAAAGTGCTATCAACACGGTAAAGTCGTAACCTGCCACAGAGACATGAAGTTCTGCTATCACAACACAGGTATGCCTTTTAGAAATTTGAAGTTGATATTGCAAGGTTGTTCTTCATCCTGCTCTGAAACTGAAAACAATAAGTGCTGCTCCACCGACAGATGTAACAAAGGTTCA

**Translated protein sequence**:

**MKREAEA**LKCYQHGKVVTCHRDMKFCYHNTGMPFRNLKLILQGCSSSCSETENNKCCSTDRCNKGS

**Number of amino acids:** 66; **Molecular weight:** 7522.65; **Theoretical pI:** 8.87

**Refolding result:** good

**I.B. solubilization solution**: 50 mM Tris-HCl, pH 9.0, 6 M Guanidine-HCl, 5 mM 2-ME

**Refolding solution**: 75 mM Tris base, 16 mM cysteine, 0.2 M NaCl

**Yield:** 0.2~0.5 mg purified product / g bacteria (wet pellet)

**Key process**: **NOT** concentrate to dry (would be hard to resolubilize if concentrate to dry), instead, dialyze the retention against 20 mM sodium acetate, pH 5.0 to remove contaminated proteins and multimeric species, which would precipitate out

**Crystallization condition**: rec-Mambalgin-1(122 mg/ml in 200 mM NH4Ac (pH 7.0)), 1:1 (v/v) with 0.1 M HEPES, pH 7.0, 32% Jeffamine M600, 0.1 M KSCN, 18 °C

**X-ray diffraction data collection:** yes

**Structural solution:** yes

**rec-Hannalgesin**

**Coding sequence**:

atgACGAAATGCTACGTTACCCCGGATGTTAAAAGCGAAACCTGCCCGGCTGGTCAAGATATTTGCTACACGGAAACCTGGTGCGATGCGTGGTGCACCAGCCGTGGCAAACGCGTCAACCTGGGTTGCGCGGCCACGTGTCCGATTGTGAAACCGGGCGTTGAAATCAAATGCTGCTCCACCGACAACTGTAACCCGTTCCCGACCCGCAAACGCCCGTAA

**Translated protein sequence**:

**M**TKCYVTPDVKSETCPAGQDICYTETWCDAWCTSRGKRVNLGCAATCPIVKPGVEIKCCSTDNCNPFPTRKRP

**Number of amino acids**: 73 Molecular weight: 8050.3 Theoretical pI: 8.36

**Refolding result:** good

**I.B. solubilization solution**: 50 mM Tris-HCl, pH 9.0, 6 M Guanidine-HCl, 5 mM 2-ME

**Refolding solution**: 75 mM Tris base, 16 mM cysteine, 0.2 M NaCl

**Yield:** 1~2 mg purified product / g bacteria (wet pellet)

**Key process**: Concentrate to dry (would efficiently remove contaminated proteins and multimeric species) and resolubilize the peptide with 20 mM sodium acetate, pH 5.0

**Crystallization condition**: rec-hannalgesin at 80 mg/ml in 200 mM NH_4_Ac (pH 7.0), 1:1 with 0.1 M Bis-Tris, 0.2 M (NH_4_)_2_SO_4_, 25% PEG3350, at 18°C

**X-ray diffraction data collection:** yes

**Structural solution:** yes

**rec-mSlurp1 (recombinant mouse Slurp1)**

**Coding sequence**:

atgTTTCGCTGCTATACCTGTGAACAACCGACGGCTATCAACTCATGTAAAAATATCGCTCAATGTAAAATGGAAGACACCGCCTGCAAAACCGTGCTGGAAACGGTTGAAGCGGCCTTTCCGTTCAACCATTCCCCGATGGTCACCCGTAGCTGCAGCTCTAGTTGTCTGGCAACGGATCCGGACGGCATTGGTGTTGCGCACCCGGTGTTCTGCTGTTTCCGTGACCTGTGTAACTCTGGTTTTCCGGGCTTTGTGGCGGGCCTGTAA

**Translated protein sequence**:

**M**FRCYTCEQPTAINSCKNIAQCKMEDTACKTVLETVEAAFPFNHSPMVTRSCSSSCLATDPDGIGVAHPVFCCFRDLCNSGFPGFVAGL

**Number of amino acids:** 89; **Molecular weight:** 9594.04; **Theoretical pI:** 5.47

**Refolding result:** good

**I.B. solubilization solution**: 50 mM Tris base, 8 M urea, 5 mM 2-ME

**Refolding condition**: 50 mM Tris-HCl (pH 9.0), 4 mM cysteine

**Key process**: concentrate NOT to dry, dialyze against 10 mM HEPES, pH 7.5 and purified with mono Q column

**Crystallization condition**: 128 mg/ml with equal ­­­­­0.8 M Succinic acid pH 7.0 at 4 °C

**X-ray diffraction data collection:** No

**rec-hSlurp1(recombinant human Slurp1)**

**coding sequence**:

atgCTGAAATGCTACACCTGCAAAGAACCGATGACCTCTGCTTCTTGCCGTACCATCACCCGTTGCAAACCGGAAGACACCGCTTGCATGACCACCCTGGTTACCGTTGAAGCTGAATACCCGTTCAACCAGTCTCCGGTTGTTACCCGTTCTTGCTCTTCTTCTTGCGTTGCTACCGACCCGGACTCTATCGGTGCTGCTCACCTGATCTTCTGCTGCTTCCGTGACCTGTGCAACTCTGAACTGTAA

**Translated protein sequence**:

**M**LKCYTCKEPMTSASCRTITRCKPEDTACMTTLVTVEAEYPFNQSPVVTRSCSSSCVATDPDSIGAAHLIFCCFRDLCNSEL

**Number of amino acids**: 82; **Molecular weight**: 8984.3; **Theoretical pI**: 5.15

**Refolding Result:** good

**I.B. solubilization solution**: 50 mM Tris base, 8 M urea, 5 mM 2-ME

**Refolding condition**: 75 mM Tris Base, 0.2 M NaCl

**Key process**: 1. Fusion of 6 x Histidine tag would significantly reduce refolding efficiency; 2. **NOT** concentrate to dry after refolding, dialyze against 10 mM HEPES, pH 7.5 and purified with mono Q column

**Crystallization condition**: no crystal obtained

**rec-mPate-B(recombinant mouse Pate B)**

**Coding sequence**:

atgCTGATCTGCAACTCTTGCGAAAAATCTCGTGACTCTCGTTGCACCATGTCTCAGTCTCGTTGCGTTGCTAAACCGGGTGAATCTTGCTCTACCGTTTCTCACTTCGTTGGTACCAAACACGTTTACTCTAAACAGATGTGCTCTCCGCAGTGCAAAGAAAAACAGCTGAACACCGGTAAAAAACTGATCTACATCATGTTCGGTGAAAAAAACCTGATGAACTTCctcgagCACCACCACCACCACCACTGA

**Translated protein sequence**:

**M**LICNSCEKSRDSRCTMSQSRCVAKPGESCSTVSHFVGTKHVYSKQMCSPQCKEKQLNTGKKLIYIMFGEKNLMNFLEHHHHHH

**Number of amino acids**: 84; **Molecular weight:** 9683.22; **Theoretical pI:** 9.08

**Refolding Result:** good

**I.B. solubilization solution**: 5 mM imidazole, 6 mM Guanidine-HCl, 10 mM 2-ME

**Refolding condition**: 50 mM Tris-HCl, pH 9

**Yield: > 1**mg purified product / g bacteria (wet pellet)

**Key process**: 1. Fusion of 6xHistidine tag did not significantly reduce refolding efficiency; 2. Concentrating to dry after refolding would efficiently remove contaminated proteins and multimeric species) and resolubilize the peptide with 20 mM sodium acetate, pH 5.0 and purified with mono S column

**Crystallization condition**: no crystal obtained
