# Supplementary material for: Scalable production of recombinant three-finger proteins: from inclusion bodies to high quality molecular probes
Source: Microb Cell Fact. 2024 Feb 12;23:48. doi: 10.1186/s12934-024-02316-1 (PMC10860255; doi:10.1186/s12934-024-02316-1)
Supplement: Supplementary file 5 — Additional file 5: Table S1. Solutions and media table. [file 12934_2024_2316_MOESM5_ESM.docx]

| **Solution Category** | **Solution (Medium) Name** | **Contents** | **Additional Information** |
| --- | --- | --- | --- |
| **Culture Media** | *E. coli* small scale culture and protein expression medium | 2xYT with 0.5% Glucose, with antibiotics* | volume = 0.2 x volume of the baffled flask |
|  | Base fermentation medium | 1% Yeast Extract, 1.6% Tryptone, 20 mM (NH_4_)_2_SO_4_, 0.02% Antifoam 204 (Sigma-Aldrich), autoclave sterilize, add to the following concentration with corresponding stock solutions: 1% glucose, 2 mM MgCl_2_, 50 mM phosphate buffer, and antibiotics* | volume = 0.6 x volume of the bioreactor |
| **Feed medium** | GYT | 10% glycerol, 10%Yeast extract and 10% Tryptone (GYT) | autoclave sterilize |
| **Stock solution** | 1 M Tris Base | 1 M Tris Base | pH not adjusted |
|  | 256 mM L-cysteine | 256 mM L-cysteine |  |
|  | 2 M NDSB-201 | 2 M NDSB-201 |  |
|  | 1 M Tris-HCl, pH 8.0 | 1 M Tris-HCl, pH 8.0 | pH adjusted with conc. HCl |
|  | 1 M Tris-HCl, pH 9.0 | 1 M Tris-HCl, pH 9.0 |  |
|  | 2 M L-arginine-HCl (pH 8.8) | 2 M L-arginine-HCl (pH 8.8) |  |
|  | 5 M NaCl | 1 M MgCl_2_ | autoclave sterilize |
|  | 1 M MgCl_2_ | 1 M MgCl_2_ |  |
|  | 40% Glucose | 40% Glucose |  |
|  | 1 M disodium phosphate | 1 M disodium phosphate |  |
|  | 1 M potassium dihydrogen phosphate | 1 M potassium dihydrogen phosphate |  |
|  | Ampecillin stock | 100 mg/ml Ampicillin | 0.2 μm filter sterilize |
|  | Kanamycin stock | 100 mg/ml Kanamycin |  |
|  | 1 M IPTG | 1 M IPTG |  |
| **Working solution** | Lysis buffer | 50 mM Tris-HCl (pH 8.0), 150 mM NaCl, 1% Triton X-100, 10 mM 2-mercaptoethanol (2-ME) | freshly prepared |
|  | Solubilization buffer | 50 mM Tris-HCl (pH 9.0), 6 M guanidine-HCl (or 8 M urea), and 5 mM 2-ME |  |
| **Refolding screening solution** | Refolding buffer set 1 | 75 mM Tris Base, 0.4 M NDSB-201, 0.5 M L-arginine-HCl, 0.2 M NaCl, with 0, 2, 4, 8, 16 mM L-cysteine |  |
|  | Refolding buffer set 2 | 75 mM Tris Base, 0.4 M NDSB-201, 0.5 M L-arginine-HCl, with 0, 2, 4, 8, 16 mM L-cysteine |  |
|  | Refolding buffer set 3 | 75 mM Tris Base, 0.4 M NDSB-201, 0.2 M NaCl, with 0, 2, 4, 8, 16 mM L-cysteine |  |
|  | Refolding buffer set 4 | 75 mM Tris Base, 0.4 M NDSB-201, with 0, 2, 4, 8, 16 mM L-cysteine |  |
|  | Refolding buffer set 5 | 75 mM Tris Base, 0.5 M L-arginine-HCl, 0.2 M NaCl, with 0, 2, 4, 8, 16 L-cysteine |  |
|  | Refolding buffer set 6 | 75 mM Tris Base, 0.5 M L-arginine-HCl, with 0, 2, 4, 8, 16 mM L-cysteine |  |
|  | Refolding buffer set 7 | 75 mM Tris Base, 0.2 M NaCl, with 0, 2, 4, 8, 16, 32, 64 mM L-cysteine |  |
|  | Refolding buffer set 8 | 75 mM Tris Base, with 0, 2, 4, 8, 16, 32, 64 mM L-cysteine |  |
|  | Refolding buffer set 9 | 0.4 M NDSB-201,0.5 M L-arginine-HCl, 0.2 M NaCl, with 0, 2, 4, 8, 16 mM L-cysteine |  |
|  | Refolding buffer set 10 | 0.2 M NaCl, with 0, 2, 4, 8, 16 mM L-cysteine |  |
|  | Refolding buffer set 11 | 0.5 M L-arginine-HCl, with 0, 2, 4, 8, 16 mM L-cysteine |  |
|  | Refolding buffer set 12 | 0.4 M NDSB-201, with 0, 2, 4, 8, 16 mM L-cysteine |  |
|  | Refolding buffer set 13 | 0.4 M NDSB-201,0.5 M L-arginine-HCl, with 0, 2, 4, 8, 16 mM L-cysteine |  |
|  | Refolding buffer set 14 | 0.4 M NDSB-201, 0.2 M NaCl, with 0, 2, 4, 8, 16 mM L-cysteine |  |
|  | Refolding buffer set 15 | 0.5 M L-arginine-HCl, 0.2 M NaCl, with 0, 2, 4, 8, 16 mM L-cysteine |  |

* Depends on the antibiotic resistance of the expression plasmid used
