# Supplementary material for: Scalable production of recombinant three-finger proteins: from inclusion bodies to high quality molecular probes
Source: Microb Cell Fact. 2024 Feb 12;23:48. doi: 10.1186/s12934-024-02316-1 (PMC10860255; doi:10.1186/s12934-024-02316-1)
Supplement: Supplementary file 6 — Additional file 6: Table S2. Properties and references of various TFPs we successfully refolded and purified. [file 12934_2024_2316_MOESM6_ESM.docx]

| TFP name | **Origin** | **Specificity** | **No. of disulfide bonds** | **Known crystal structure?** | **Reference** |
| --- | --- | --- | --- | --- | --- |
| αBtx | Bungarus Multicinctus | α_1_β_1_γ(ε)δ nAChR blocker | 5 | Yes | [42,66,67] |
| κBtx | Bungarus Multicinctus | α_3_β_2_ nAChR blocker | 5 | Yes | [5,6,68–71] |
| Hannalgesin | Ophiophagus hannah | NOS activator  α_1_β_1_γ(ε)δ nAChR blocker | 5 | No | [2,72] |
| Mambalgin-1 | Dendroaspis polylepis | ASIC1a blocker | 4 | Yes | [1,63–65,73] |
| MTα | Dendrosaspis angusticeps | α2B-adrenoceptor blocker | 4 | No | [74] |
| αCTX | Naja kaouthia | α_2_βγε nAChR blocker | 5 | Yes | [75,76] |
| mouse  Pate B | Mouse | phosphatidylethano- lamine phosphatidylserine? | 5 | No | [20,23,77] |
| hSlurp1 | Human | α7 nAChR? | 5 | No | [78,79] |
| mSlurp1 | Mouse | ? | 5 | No | [80,81] |
|  | | | | | |
