# Supplementary material for: Scalable production of recombinant three-finger proteins: from inclusion bodies to high quality molecular probes
Source: Microb Cell Fact. 2024 Feb 12;23:48. doi: 10.1186/s12934-024-02316-1 (PMC10860255; doi:10.1186/s12934-024-02316-1)
Supplement: Supplementary file 7 — Additional file 7: Table S3. Expression level, monomer fraction and relative amount from dilution refolding and final yield table for rTFPs. [file 12934_2024_2316_MOESM7_ESM.docx]

| rTFP Name | Expression Level^*1^  (mg/g wet pellets) | dilution refolding monomer ratio^*2^ | rel. monomer amount^*3^ | Final Yield^*4^  (mg/g wet pellets) |
| --- | --- | --- | --- | --- |
| rec-αBtx | 6.1 | 0.51 | N/A | 0.05 |
| rec-Hannalgesin | 2.8 | 0.78 | 0.74 | 1~2 |
| rec-κBtx | 4.6 | 0.74 | 0.61 | 0.1 |
| rec-MΤα | 14.7 | 0.81 | 0.63 | 1~2 |
| rec-αCTX | 2.4 | 0.78 | 0.39 | >1 |
| rec-Mambalgin | 4.2 | 0.37 | 0.96 | 0.5 |
| rec-mSlupr1 | 2.6 | N/A | N/A | N/A |
| rec-mPate B | 3.2 | N/A | N/A | >1 |
| rec-hSlurp1 | 3.5 | 0.37 | 1 | N/A |

*1: For estimation of expression level in terms of mg protein/g wet cell pellets, assume induced sample equivalent to 220 μg of wet cell pellets was loaded per lane and each protein ladder band corresponds to 0.5 μg of protein.

*2 and *3: Calculated from refolding conditions selected by eyeballing the non-reducing SDS-PAGE of the refolding screening experiment shown in Supplementary Figure 1.

*4 Purified protein amount was calculated by volume and protein concentration, which was calculated with extinction coefficient based on a.a. sequence and OD_280_
